# MiRNA-200C expression in Fanconi anemia pathway functionally deficient lung cancers

**DOI:** 10.1038/s41598-021-83884-9

**Published:** 2021-02-24

**Authors:** Wenrui Duan, Shirley Tang, Li Gao, Kathleen Dotts, Andrew Fink, Arjun Kalvala, Brittany Aguila, Qi-En Wang, Miguel A. Villalona-Calero

**Affiliations:** 1 Department of Human and Molecular Genetics, Herbert Wertheim College of Medicine, The Florida International University, Miami, FL 33199 USA; 2 Biomolecular Sciences Institute, The Florida International University, Miami, FL 33199 USA; 3 Comprehensive Cancer Center at The Ohio State University College of Medicine and Public Health, Columbus, OH 43210 USA; 4 Department of Radiation Oncology, Comprehensive Cancer Center, The Ohio State University, Columbus, OH 43210 USA; 5 Department of Medical Oncology and Therapeutics Research, City of Hope National Medical Center, Duarte, CA 91010 USA

**Keywords:** Non-small-cell lung cancer, Cancer genetics

## Abstract

The Fanconi Anemia (FA) pathway is essential for human cells to maintain genomic integrity following DNA damage. This pathway is involved in repairing damaged DNA through homologous recombination. Cancers with a defective FA pathway are expected to be more sensitive to cross-link based therapy or PARP inhibitors. To evaluate downstream effectors of the FA pathway, we studied the expression of 734 different micro RNAs (miRNA) using NanoString nCounter miRNA array in two FA defective lung cancer cells and matched control cells, along with two lung tumors and matched non-tumor tissue samples that were deficient in the FA pathway. Selected miRNA expression was validated with real-time PCR analysis. Among 734 different miRNAs, a cluster of microRNAs were found to be up-regulated including an important cancer related micro RNA, miR-200C. MiRNA-200C has been reported as a negative regulator of epithelial-mesenchymal transition (EMT) and inhibits cell migration and invasion by promoting the upregulation of E-cadherin through targeting ZEB1 and ZEB2 transcription factors. miRNA-200C was increased in the FA defective lung cancers as compared to controls. AmpliSeq analysis showed significant reduction in ZEB1 and ZEB2 mRNA expression. Our findings indicate the miRNA-200C potentially play a very important role in FA pathway downstream regulation.

## Introduction

**Figure 1:**
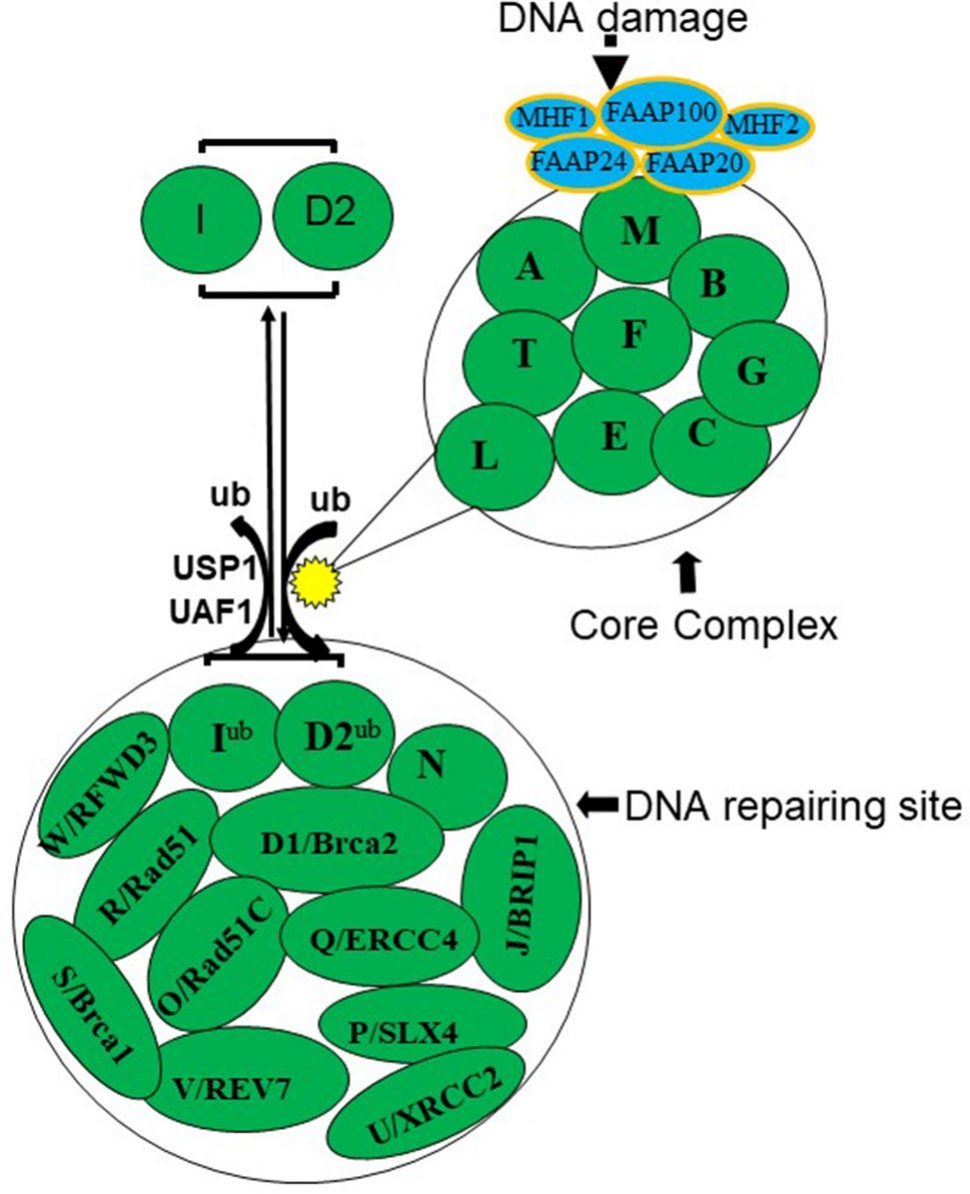
The Fanconi anemia (FA) pathway. The FA pathway consists of Core Complex proteins and other downstream FA proteins involved in DNA repair process. The FA Core Complex consists of FANCA, B, C, E, F, G, L, M and T (UBET2) along with other additional proteins that associate with the FA Core Complex, including FAAP100, FAAP24, FAAP20, and the histone fold dimer proteins MHF1 and MHF2. The FA Core Complex activates FANCD2 and FANCI by mono-ubiquitinating the protein as a response to DNA damage. The activated FANCD2–I heterodimeric proteins are subsequently transported to sub-nuclear foci, thought to be the sites of DNA repair, which also contain FA downstream proteins including the DNA repair proteins FANCD1(BRCA2), D2, I, J, N, O, P, Q, R, S (BRCA1), U, V and W.

FA is an autosomal recessive disorder (with an exception of the complementation group B, being X-linked) most commonly associated with DNA damage, progressive bone marrow failure (BMF) and escalated risks of cancer^1–5^. This complex heterogeneous disease is linked to mutations in 22 genes^2^ identified to date (Fig. 1), referred to as FA subtypes A, B, C, D1/BRCA2, D2, E, F, G, I, J/BRIP1, L, M, N/PALB2, O/RAD51C, P/SLX4, Q/ERCC4, R/RAD51, S/BRCA1, T/UBE2T^2,3^, U/XRCC2^4^, V/REV7^5^ and W/RFWD3^6^. Normal function of these genes is necessary for the FA pathway to operate mediating homologous recombination, interstrand cross-linked (ICL) repairs, and other DNA repair functions^7–10^. Cells with FA pathway deficiency are hypersensitive to DNA damage agents such as cisplatin and mitomycin C (MMC). It has also been shown that patient tumor with germ line FA gene deficiency (e. g. BRCA1, BRCA2, Rad51C) are sensitive to DNA damaging agents and inhibitors of other repair pathways such as PARP inhibitors^11–13^.

The FA pathway consists of Core Complex proteins and other downstream FA proteins involved in DNA repair process^1^. The FA Core Complex consists of FANCA, B, C, E, F, G, L, M and T (UBET2) along with other additional associated proteins, including FAAP100, FAAP24, FAAP20, and the histone fold dimer proteins MHF1 and MHF2 ^10,14,15^.

The FA Core Complex serves as a nuclear E3 ubiquitin ligase for two other very important FA proteins, FANCD2 and FANCI^14–19^. The activated FANCD2–FANCI heterodimeric proteins are subsequently transported to sub-nuclear foci, thought to be the sites of DNA repair, which also contain FA pathway downstream proteins including the DNA repair proteins FANCD1 (BRCA2), J, N, O, P, Q, R, S, U, V and W^1–6,10,14,15,20–22^. De-ubiquitination of FANCD2 by the ubiquitin-specific protease 1 (USP1), FANCR(RAD51) by FANCW(RFWD3) result in these protein inactivation and release from the sites of DNA repair ^21–24^ (Fig. 1). Since the Core Complex proteins ubiquitinate and activate FANCD2, thus the FANCD2 protein is the key effector protein in the FA pathway. FANCD2 is converted from a small (S) to a large (L) form by mono-ubiquitination during S phase of cell cycle or following induction of DNA double-strand breaks or interstrand crosslinks^14–16^. Any mutation or epigenetic change that disrupts components of the Core Complex also abrogates its E3 ligase function, leading to defective FA pathway.

In order to evaluate functionality of the FA pathway, we developed a Triple-Staining Immunofluorescence method (FATSI), which is able to detect FANCD2 foci formation or lack of formation in paraffin embedded tumor tissues^8,13^. In one study, a total of 139 non-small cell lung cancer formalin-fixed paraffin embedded (FFPE) tumor samples were screened; 104 were evaluable for FANCD2 foci status. Eighty-one of the 104 (81/104, 78%) evaluable tumors were found FANCD2 foci positive and 23 (22%) were foci negative^9^. In a separate study, we analyzed total of 643 human solid tumors and one hundred eighty-five of 643 (185/643, 28.7%) screened patients were FANCD2 foci negative^13^, documenting a 19.7% of lung cancers being functionally deficient.

Recent studies showed that MicroRNA (miRNA) expression is associated with DNA repair pathways^24^. MiRNAs are small, 18 to 25 nucleotide noncoding RNAs, which regulate target genes mRNA levels and direct regulation of protein synthesis of target genes^25^. MiRNAs play important endogenous roles in biological pathways of mammals and multicellular organisms via targeting mRNA^26–29^. Increasing evidence suggests that microRNAs can function as either oncogenes or tumor suppressor genes^30^. They derive from larger precursors, which form stem-loop structures with the mature miRNA as one arm of the precursor hairpin released by ribonuclease III (RNase III)^31^.

MiRNAs are critical to cancer development due to their regulatory effects on genes involved in apoptosis, cell cycle progression, differentiation, migration, metabolism, epithelial-mesenchymal transition and tumor cell invasion and metastasis^26–29^. Among these regulations, the most common way is via translational silencing of gene expression via suppression or degradation by binding to target mRNA at the 3′ untranslated region UTR through base pairing^27,32,33^. A single miRNA may target several hundred mRNAs and can function as oncogenes or tumor suppressors ^26,32–34^. Many studies have shown that certain miRNAs can serve as potential cancer biomarkers for diagnosis or prognosis^27,34–36^.

We report herein results of our evaluation of regulation and expression of miRNAs in FA pathway deficient cancers and discuss potential implications on their use as predictive or prognostic biomarkers.

## Methods

### Tissue samples and FA triple-stain immunofluorescence analysis (FATSI)

Human non-small cell lung cancer (NSCLC) samples were obtained from the Tissue Procurement Shared Resources of the Ohio State University Comprehensive Cancer Center and The Cooperative Human Tissue Network, Midwestern Division at The Ohio State University, after institutional review board (IRB) approval. A total of 29 non-small cell lung cancer samples, including 16 FANCD2 foci negative and 13 foci positive tumors, and matched non-tumor tissue samples were analyzed.

FFPE tumor tissue was cut at 4 microns, placed on positively charged slides, and stained with hematoxylin–eosin. Additional sections for immunofluorescence staining were processed as described previously^8,9^. Briefly, slides were laced in a 60 °C oven for 1 h, cooled, deparaffinized, and rehydrated through xylenes and graded ethanol solutions to water in standard fashion. The tissue sections were incubated with a primary antibody cocktail of rabbit polyclonal FANC-D2 antibody (Novus Biologicals, Littleton, CO, Catalog #: NB100-182) at a dilution of 1:1000 and a monoclonal anti-Ki67 mouse antibody (Dako, Carpenteria, CA, USA, Catalog #: M7240) at a dilution of 1:150 for 1 h at room temperature. Sections then were co-incubated with a secondary antibody fluorescein isothiocyanate (FITC) conjugated to anti-rabbit immunoglobulin (Ig)G (Novus Biologicals, Littleton, CO, Catalog #: NBP2-30342F) and Alexa Fluor 594 donkey anti-mouse IgG (Invitrogen, Carlsbad, California, Catalog #: A32744 ) at 1:1000 for 1 h at room temperature. All rinses were performed on the auto-stainer with TBS-T. The sections were mounted on glass slides using a DAPI-containing embedding medium (Vysis Dapi 1, Abbott Laboratories, Downers Grove, Ill). The slides were analyzed under a Nikon (Tokyo, Japan) E-400 fluorescence microscope^8^.

### FANCD2 defective cells

**Figure 2:**
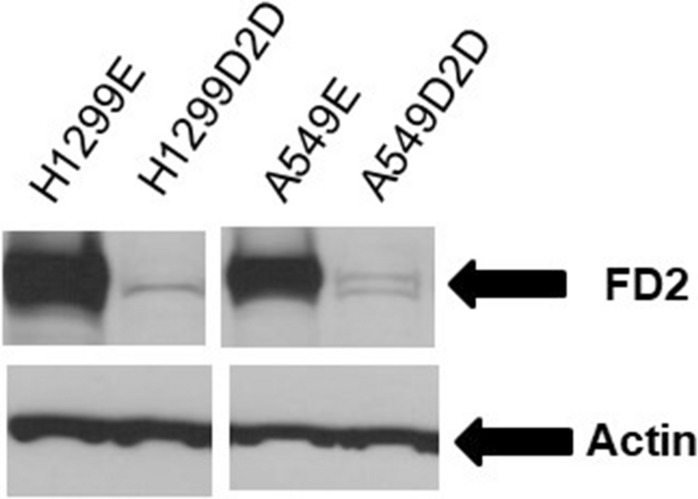
Western blot analysis of FANCD2 protein expression. Non-small cell lung cancer cells H1299 and A549 were transduced with FANCD2-specific shRNA-expressing or empty vector and puromycin-resistant lentiviral particles. The transduced cells were selected in 4 ug/ml puromycin to create stably transduced cells with reduced FANCD2 expression, the H1299D2D and A549D2D. H1299E and A549E were transduced with empty lentiviral particles. FANCD2 protein expression was confirmed by western blot analysis.

The FANCD2 foci defective cells, A549D2D and H1299D2D were created by knocking down FancD2 expression as described previously^9^. Briefly, non-small cell lung cancer cells A549 and H1299 were plated 24 h prior to transduction and when the cells reached 60% confluence, cells were transduced with FANCD2-specific shRNA-lentiviral particles conferring resistance to puromycin. Control shRNA lentiviral particles were also transduced into the cells (Santa Cruz Biotechnology Inc., Santa Cruz, CA, Catalog#: SC-35356-V) according to the manufacturer’s protocol. One day after incubation in medium containing polybrene agent, these transduced cells were transferred to a dish that contains normal growth medium. The transduced cells were selected in 4ug/ml puromycin. To create stably transduced cells, 100–200 transduced cells were cultured in a 100 mm dish, and medium was replaced with fresh puromycin-containing medium every 3 days, until resistant colonies were identified. Twenty colonies were picked for each cell line, and then the colonies were expanded. Successful FANCD2 knockdown was confirmed by western blot (Fig. 2). The FA effective cells, A549E and H1299E were A549 and H1299 cells transfected with empty vectors. SV40 transformed empty retroviral vector transduced human FANCD2 defective fibroblasts PD20RV (FancD2 deletion) and their wild-type FANCD2 transduced counterparts PD20D2 (FANCD2 effective)^37^ were obtained from the Oregon Health and Science University Fanconi Anemia Cell Repository using as control cells.

### Western immunoblot analysis

Western immunoblot analysis was performed as described previously^9^. Briefly, cells were digested with the cell lysis buffer (Cell Signaling Technology Inc., Danvers, MA, Catalog #: 9803). Protein concentrations were evaluated using the Bradford reagent (Bio-Rad, Hercules, CA, USA). One hundred micrograms of total protein was loaded onto NuPAGE 4–12% Bis–Tris Gel (Invitrogen, Carlsbad, CA, USA). Protein on the gels was electro-transferred onto nitrocellulose membranes and blocked with blocking buffer (5% of non-fat milk, 500 mM of NaCl, 20 mM Tris, and 0.1% Tween 20). The membranes were incubated with primary anti-bodies at 4 °C overnight. After washing with TBS-T (blocking buffer without milk) five times, 10 min each, the membranes were incubated with anti-mouse IgG (Amersham Pharmacia Biotech, Piscataway, NJ, USA, Catalog #: NA931) or anti-rabbit IgG horse radish peroxidase linked to whole secondary antibodies (Amersham Pharmacia Biotech, Piscataway, NJ, USA, Catalog #: NA934) at room temperature for 1 h. A chemiluminescent detection system (ECLwestern blotting detection reagents, GE) was used to detect the secondary antibody. Finally, the membranes were exposed to x-ray films. Antibodies used were rabbit polyclonal FANCD2 antibody (NovusBiologicals, Littleton, CO, USA, Catalog #: NB100-182) and anti-Actin monoclonal (Sigma, St. Louis, MI, USA, Catalog #: A2228).

### RNA extraction

Total RNA was extracted from cell and tissue samples via TRIzol Reagent isolation following the protocol supplied by the manufacturer (Thermo Fisher Scientific, Waltham, MA, Catalog #:15,596,026). RNA samples were treated with DNase to remove contaminating DNA and stored in –80 °C freezer.

### NanoString nCounter miRNA expression

nCounter miRNA assays are highly sensitive and based on direct molecular barcoding and digital detection of targeted RNA molecules of interest though target-specific color-coded probe pairs and does not require conversion of RNA to cDNA by reverse transcription via PCR. Probe pairs have a reporter probe carrying signals on 5′ end, and a capture probe with biotin ion at 3′. Hybridization washes away access probes and allows for RNA probe complexed to be aligned and immobilized for data collection of absolute miRNA expression^38^. Based on the miRNA Expression Assay User Manual from NanoString Technologies, RNA samples were normalized to 33 ng/uL in DEPC water. An annealing master mix was created (13µL Annealing Buffer, 26µL nCounter miRNA Tag Reagent, and 6.5 µL 1:500 miRNA Assay Controls dilution). 3.5 µL of the master mix was aliquoted to 12 × 0.2 mL strip tubes with 3µL (100 ng) RNA sample and cycled with Annealing Protocol. 2.5 µL Ligation master mix (19.5 VL PEG, 13µL Ligation buffer) was added to each tube at 48 °C in thermocycler and incubated for 5 min before adding 1.0uL ligase for ligation protocol. 1.0 µL of Ligation Clean-Up Enzyme was added to each reaction and the tubes were removed from heat to be spun down before returning to cycler for purification protocol. DEPC (40µL) was added to each sample for hybridization. 20 µL master mix (130 µL Reporter Code set, 130 µL hybridization buffer) were added to each clean tube with 5uL aliquot of miRNA sample in DEPC. 5uL of Capture probe set was added to each tube before placing in preheated 65 °ºC thermocycler and incubated for 12 h. The samples where then placed in post-hybridization processing with nCounter Prep Station immediately after removal from thermocycler. Background correction factors were added to miRNAs, technical normalization using positive control, and biological normalization was performed for absolute miRNA expression.

### Quantitative real time PCR

Quantitation of miRNAs was carried out using TaqMan microRNA assays (Applied Biosystems, Foster City, CA) as described previously^39^. MicroRNA-200C expression kit (catalog # 4,427,975, Assay ID: 002,286) and FANCD2 gene expression kit (Catalog # 4,331,182, Assay ID: Hs00276992_m1) were purchased from the ThermoFisher Scientific (ThermoFisher Scientific /Applied Biosystems, Foster City, CA). Reverse transcription was performed by using TaqMan MicroRNA Reverse Transcription Kit (Applied Biosystems, Foster City, CA). The PCR amplification was conducted in 25 μl reaction using the TaqMan Universal PCR Master Mixture, No AmpErase UNG kit (Applied Biosystems, Foster City, CA) following the protocol provided by the manufacturer. Real-time PCR was carried out in a 96-well plate using a Bio-Rad CFX96 system. Every sample was done in triplicate and each reaction was repeated at least once to ensure reproducibility. The PCR cycle number at threshold (CT) was used for the comparison. The relative quantitative method was used for the quantitative analysis^40^. Resulting data was quantified and normalized using RNU48 as control.

### Ion AmpliSeq RNA gene expression

Reverse transcription was performed on RNA samples with 1 µL 10X Superscript III Enzyme Mix, 5X VILO RT Reaction Mix, and Nuclease-free water to 10 µL for each sample to obtain cDNA. Targets were amplified by adding 4uL 5X Ion AmploSqe HiFi Master Mix, 4 µL 5X Ion RNA panel, Nuclease-free water, and cDNA for a total of 20 µL. The reaction was run according to AmplisSeq RNA Library Kit User Guide from Thermofisher. Primer sequences were partially digested with addition of 2 µL FuPa Reagent and cycle accordingly before ligation to the amplicons and purification. The Library was then amplified and quantified for resulting gene expression results tissue derived RNA samples.

### Cell viability analysis

The cell viability analysis was described previously^9,39^. Five thousand FA defective and control lung cancer cells from each line (H1299E/H1299D2D, A549E/A549D2D), were seeded in each well of a 96-well plate. Cells were cultured in RPMI 1640 medium with 10% fetal bovine serum and 1% penicillin/streptomycin in a humidified incubator (37 °C, 5% of CO2) for 48 h. Dimethylthiazolyl-2-5-diphenyltetrazolium bromide (MTT) dye solution (Sigma, St. Louis, MO, USA) was added into the 96-well plate for 4 h, and the treatment terminated by adding stop solution (isopropanol with 0.04 N HCl). Absorbance at 570 nm wavelength was recorded using a Bio-Rad micro plate reader (Bio-Rad Laboratories, Inc., Hercules, CA). Each cell line was repeated in quadruplicate on same plate and each experiment was repeated at least once to ensure reproducibility. An averaged absorbance of blank values (containing all reagents except cells) was subtracted from all absorbance to yield corrected absorbance. The relative absorbance of each sample was calculated by comparing the average of corrected absorbance with an average of corrected control^9,39^.

## Results

### miRNAs expression by NanoString analysis

RNA samples isolated from the PD20RV, PD20D2, A549E, A549D2D, H1299E and H1299D2D cells as well as two FANCD2 foci negative human non-small cell lung cancers and matched non-tumor tissues, were used for miRNA expression analysis by NanoString nCounter count. After background correction and data normalization, 734 different miRNAs were detected, and fold change was calculated based on FANCD2 deficiency over control samples. A cluster of 10 miRNAs were found to be consistently up-regulated by at least twofold in two of the three cell lines (A549, H1299 and PD20), and a cluster of 3 miRNAs were found to be down regulated by at least twofold in two of the three groups in FANCD2 foci deficient samples as compared to their respective non deficient control samples (Table 1). Among all these miRNAs, miRNA-200C had consistently higher expression (6.9 fold on average) in FANCD2 foci deficient cells compared to matched control cells (Table 1). In addition, miRNA-200C expression was 22.02 folds higher in average in two FANCD2 foci defective lung cancer tissues comparing to expression of this miRNA in matched non-tumor tissues.

**Table 1:** miRNAs expression in Fanconi anemia pathway deficient cells.

Detector	Sequence	Fold change in H1299 (deficient/efficient)	Fold change in A549 (deficient/efficient)	Fold change in PD20 (deficient/efficient)
hsa-miR-20a + hsa-miR-20b	uaaagugcuuauagugcagguag(20a) caaagugcucauagugcagguag(20b)	− 1.70	− 5.58	− 25.67
hsa-miR-129-3p	aagcccuuaccccaaaaagcau	1.32	− 2.39	− 3.07
hsa-miR-626	agcugucugaaaaugucuu	2.02	− 2.33	− 2.20
hsa-miR-1288	gcagaucaggacuguaacucacc	− 1.16	2.51	2.37
hsa-miR-26a	uucaaguaauccaggauaggcu	− 1.21	7.40	370.36
hsa-miR-143	ggugcagugcugcaucucuggu	2.38	− 1.01	2.52
hsa-miR-921	cuagugagggacagaaccaggauuc	2.52	− 1.44	2.16
hsa-miR-184	uggacggagaacugauaagggu	2.46	2.03	− 1.53
hsa-miR-567	aguauguucuuccaggacagaac	2.10	3.34	− 1.14
hsa-miR-873	gcaggaacuugugagucuccu	1.56	2.79	2.16
hsa-miR-1282	ucguuugccuuuuucugcuu	2.07	4.18	1.68
hsa-miR-933	ugugcgcagggagaccucuccc	2.21	2.23	1.44
hsa-miR-200c	cgucuuacccagcaguguuugg	13.52	1.44	5.76

### Quantitative real time PCR analysis of miRNA-200C expression

**Figure 3:**
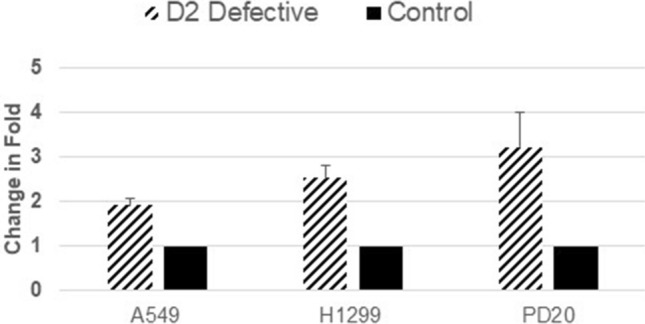
Evaluation of miRNA-200C expression with TaqMan microRNA real time PCR analysis in the FANCD2 defective and control cells. FANCD2 defective cells H1299D2D, A549D2D and PD20RV as well as matched control cells H1299E, A549E and PD20D2 cells were cultured and total RNA samples were isolated. Quantitation of miRNA expression was carried out using TaqMan real time polymerase chain reaction (PCR). Primers and probes used for the real time PCR analysis were obtained from Applied Biosystems (Foster City, CA). Real-time PCR was carried out in a 96-well plate using a Bio-Rad CFX96 system. Each sample was done in triplicate and each reaction was repeated at least once to ensure reproducibility. Resulting data were quantified and normalized using RNU48 as control.

To validate the results from NanoString analysis, quantitative PCR was used with the same cell lines along with 29 tumor and non-tumor pairs (13 FANCD2 foci positive, 16 FANCD2 foci negative). TaqMan miRNA-200C assay was employed along with RNU48 as an internal control. As depicted in Fig. 3, FANCD2 knock-down A549 cells (A549D2D) showed significant overexpression of miRNA-200C compared to the matching counterpart control A549E cell lines (p = 1.068e−3). Similarly, H1299 FANCD2 knockdown (H1299D2D) cells showed significant (p = 7.984e−3) overexpression of miRNA-200C as comparing to control cells H1299E. In PD20 (FANCD2 deficient) fibroblasts cell lines, FANCD2 was transduced into the cells to generate PD20D2. Our results indicated that miRNA-200C was also significantly overexpressed in the FANCD2 deficient PD20RV (empty vector) cell as compared to transduced wild type FANCD2 PD20D2 cell (p = 3.325e−3).

**Figure 4:**
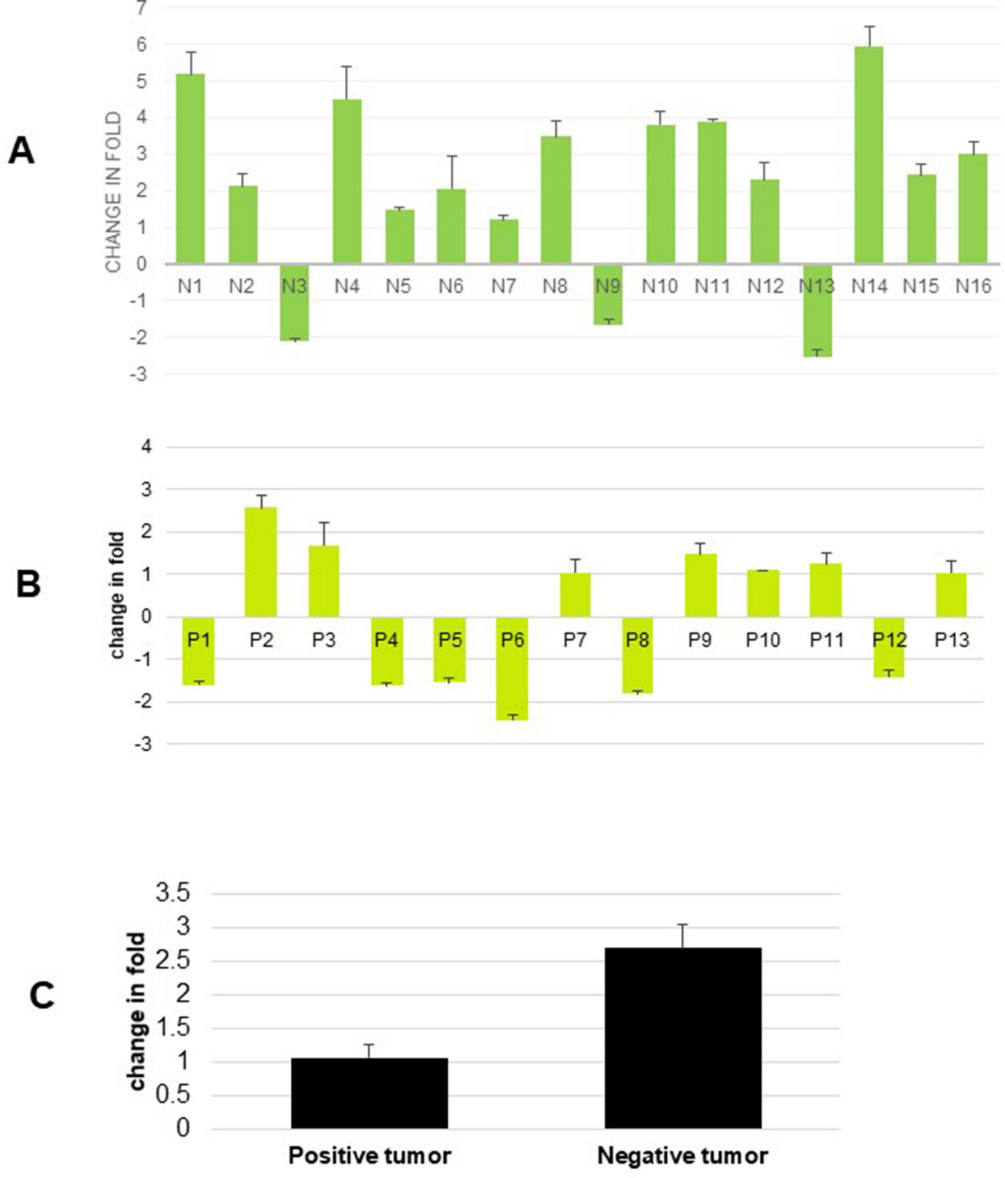
TaqMan real time PCR analysis of miRNA-200C expression in the FANCD2 foci defective and foci positive lung tumor tissues. TaqMan real time PCR was employed for analyzing miRNA-200C in FANCD2 foci positive and foci negative tumor samples. Out of the 16 FANCD2 foci negative tumors, the miRNA-200C was increased in 13 (81%) cases as compared to matched non-tumor lung tissue (**A**). Comparably, among 13 FANCD2 foci positive tumor samples, miRNA-200C was increased in 7(54%) cases (**B**). The average of fold change on miRNA-200C in the foci negative samples was 2.7 indicating overexpression in the foci negative tumors compared to matched non-tumor lung tissue. Comparably the averaged fold change in 13 foci positive tumors was nearly 1 which indicates miRNA-200C expression does not vary significantly between tumor and non-tumor counterpart for FANCD2 foci positive tumors (**C**).

In 13 FANCD2 foci positive tumor samples, miRNA-200C expression did not vary significantly between tumors and adjacent non-tumor samples. In contrast on the 16 FANCD2 foci negative tumors, significant overexpression was observed in tumors as comparing to non-tumor tissue. After normalization of the human tumors with their respective non-tumor tissue, a significant overexpression (p = 1.848e−3) of miRNA-200C in foci negative tumors was observed compared to the FANCD2 foci positive tumors as depicted in Fig. 4A–C.

### AmpliSeq analysis of miRNA-200C target genes

**Figure 5:**
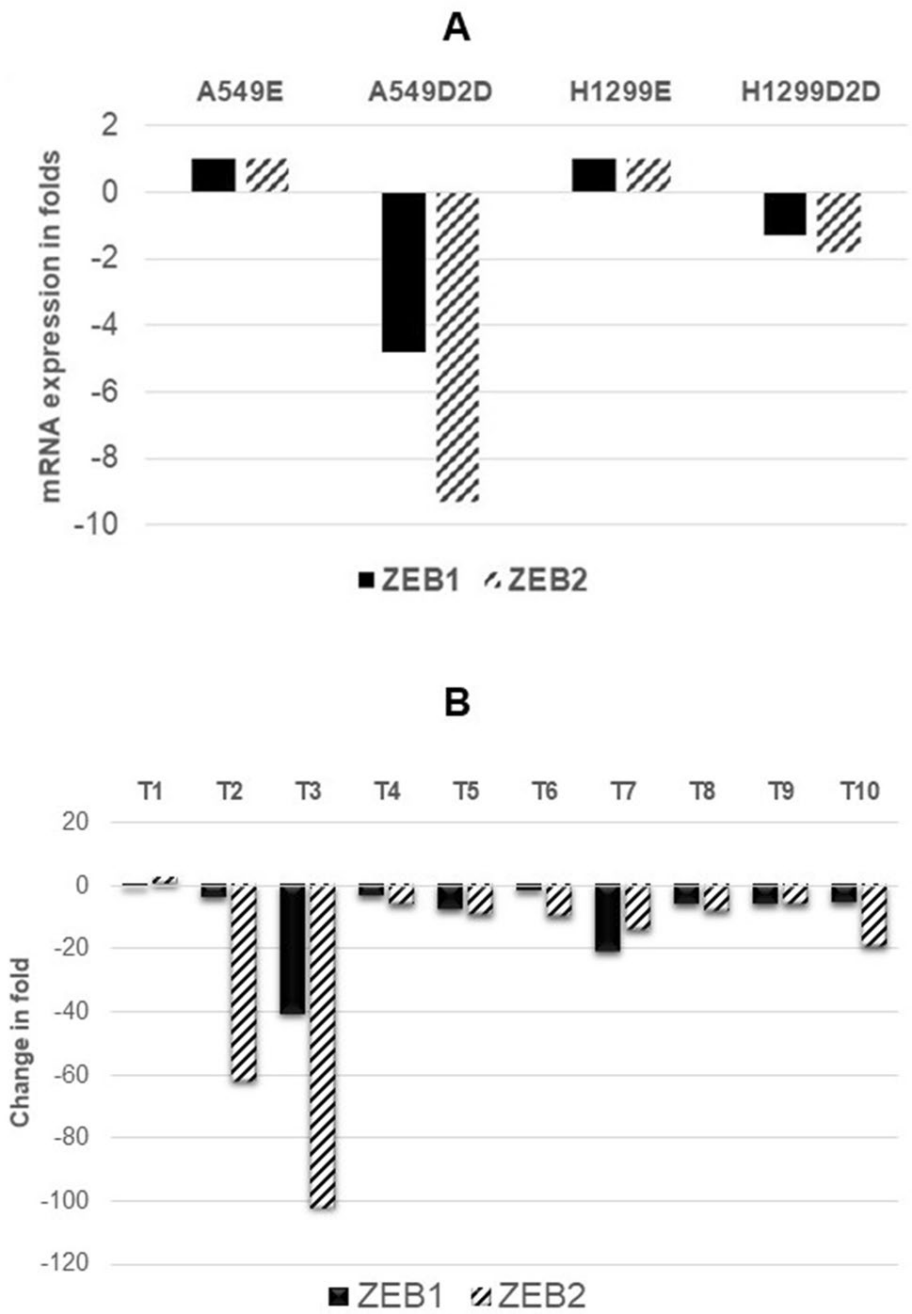
AmpliSeq analysis of ZEB1 and ZEB2 Gene RNA Expression. AmpliSeq analysis was used to analyze FANCD2 knockdown lung cancer cells and 10 FANCD2 foci negative tumor samples and matched non-tumor lung tissues in ZEB1, and ZEB2 expression levels. Both ZEB1 and ZEB2 are miRNA-200C downstream genes. Increased miRNA-200C expression in FANCD2 deficient samples would impact on expression of its target genes. A: FANCD2 knockdown lung cancer cells. B: Among the 10 FANCD2 foci negative tumor samples and matched non-tumor lung tissues (non-tumor control value is 1), 100% showed downregulation of ZEB1 and 90% for ZEB2 compared to matched non-tumors.

Previous studies have demonstrated that endogenous miRNA-200C suppresses EMT by regulating cell adhesion through targeting the E-cadherin transcriptional repressors ZEB1 and ZEB2^41,42^. AmpliSeq data from FANCD2 knockdown lung cancer cells and 10 FANCD2 foci negative tumor samples were used with a gene expression panel with specific interest in ZEB1, and ZEB2 expression levels. Both ZEB1 and ZEB2 mRNA expressions were reduced in FANCD2 knockdown lung cancer cells (Fig. 5A). All 10 tumor samples (100%) showed reduced mRNA expression in ZEB1, and 9 out of 10 samples (90%) showed reduction in ZEB2 expression as compared to matched non-tumors (Fig. 5B).

### Number of viable cells was reduced in the FANCD2 knockdown lung cancer cells A549D2D and H1299D2D

**Figure 6:**
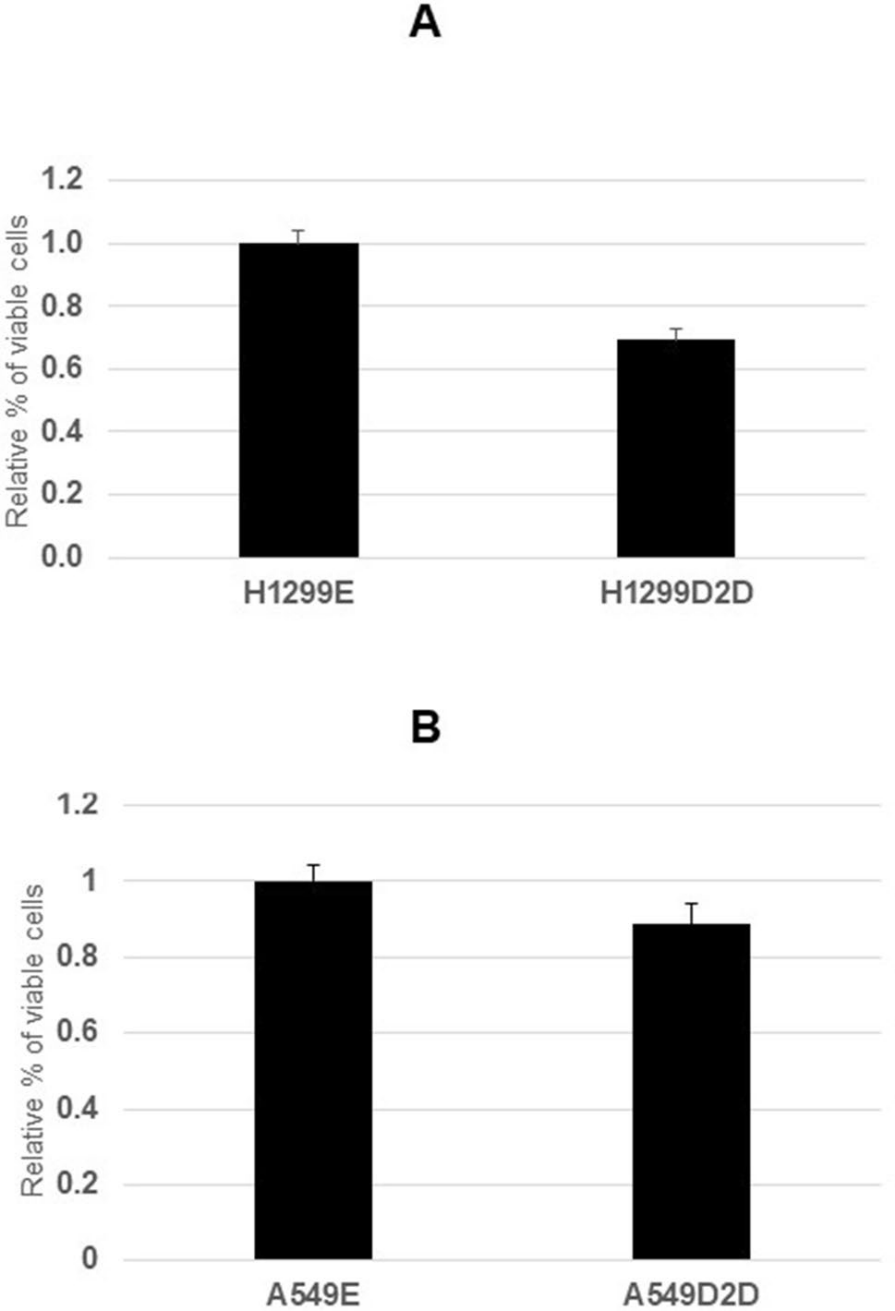
Cell viability of the FANCD2 knockdown lung cancer A549D2D and H1299D2D cells. Cells were cultured in RPMI 1640 medium with 10% fetal bovine serum and 1% penicillin/streptomycin in a humidified incubator (37 °C, 5% of CO_2_) for 48 h. Each cell line was repeated in quadruplicate. Absorbance at 570 nm wavelength was recorded using a micro plate reader. An averaged absorbance of blank values (containing all reagents except cells) was subtracted from all absorbance to yield corrected absorbance. The relative absorbance of each FANCD2 knockdown cell was calculated by comparing the average of corrected absorbance with an average of corrected absorbance of control cell. Each value presented in this figure was an average value obtained from 4 measurements. The error bars show standard deviation. (**A**) H1299D2D /H1299E, (**B**) A549D2D/A549E.

To investigate the influence of FANCD2 knockdown in cell proliferation, we investigated viability of the FANCD2 knockdown lung cancer cells (A549D2D and H1299D2D) using MTT assay. The number of viable cells was reduced in the FANCD2 knockdown cells, A549D2D and H1299D2D as comparing to their counterparts the A549E and H1299E respectively (Fig. 6). There were 70% viable cells in the H1299D2D comparing to H1299E, and 88% viable cells in the A549D2D comparing to A549E cell. These results indicate the number of viable cells was reduced in the FANCD2 knockdown lung cancer A549D2D and H1299D2D cells.

## Discussion

MiRNA-200C was found to have tumor-suppressive properties, including inhibition of the epithelial-mesenchymal transition (EMT), and invasion and metastasis in several cancers^35,42–46^. Furthermore, miRNA-200C had also been identified as a prognostic biomarker for NSCLC^47^. Ectopic overexpression of miRNA-200C down regulates p-EGFR and p-AKT and increases radiosensitivity in several cancer cell lines^45^.

Herein we report that miRNA-200C is an important FANCD2 downstream regulator of cell proliferation and cancer cell metastasis. Based on our study, miRNA-200C was upregulated in the FANCD2 foci deficient non-small cell lung cancer cells and tumor tissues. Both, NanoString nCounter data and real time PCR analysis showed overexpression of miRNA-200C in FANCD2 deficient samples compared to their non-deficient counter parts. This is the first study that shows correlation between the Fanconi Anemia pathway and miRNA expression in lung cancer cells and tumor tissues. Furthermore, when using FANCD2 knockdown lung cancer cells and tumor samples with high miRNA-200C expression, we found that the expression of ZEB1 and ZEB2 were drastically down regulated. This inverse relationship correlates with previous studies showing that miRNA-200C inhibits epithelial-mesenchymal transition, invasion, and migration of lung cancer by targeting ZEB1 and ZEB2 genes^35,44–49^.

A recent study showed expression levels of miR-200C was no significant change in human non-small cell lung cancers (NSCLCs) as it is compared to normal adjacent tissues by real-time PCR^50^. In current study, the average of fold change on miRNA-200C in the 16 FANCD2 foci negative non-small cell lung cancer was 2.7 indicating overexpression in the foci negative tumors compared to matched non-tumor lung tissue. However, the averaged fold change on miRNA-200C in 13 FANCVD2 foci positive non-small cell lung cancers was nearly 1 which indicates miRNA-200C expression was no difference between tumor and non-tumor counterpart (Fig. 4A,B). Since the lung cancers reported in the Nourmohammadi et al.^50^ were non-FA-associated NSCLC, the miRNA-200C was no significant change. In contrast, the NSCLC samples in the FANCD2 foci negative cohort were FA defective tumors in the current study. In these FANCD2 foci negative tumors, miRNA-200C was upregulated compared to matched non-cancer tissues. Our results suggest that FA deficiency upregulates the miRNA-200C expression in NSCLC.

In A459 cells overexpressing miR-200C resulted in downregulation of EMT through downregulating N-cadherin and upregulating E-cadherin. Furthermore miR-200C has also been shown to significantly reduce cell invasion and migration through inhibition of ZEB2 expression^44^. It has also been reported that downregulation of miR-200C resulted in decreased E-cadherin through upregulation of ZEB1^51^. Reduction in E-cadherin through ZEB1 leads to increase in cell motility; leading to epithelial mesenchymal transition (EMT). In addition, miR-200C overexpression significantly accelerates cell cycle arrest at G0/G1 phase, inhibits cell proliferation, and induces apoptosis in A549 cells, possibly by activating the p53/p21 pathway^35^. Thus, the FANCD2 may regulate cell cycle and metastasis indirectly through the regulation of miRNA-200C expression.

We have also investigated the viability of the FANCD2 knockdown lung cancer cells (A549D2D and H1299D2D) using MTT assay. We found the number of viable cells was reduced in the FANCD2 knockdown cells A549D2D and H1299D2D as comparing their counterparts the A549E and H1299E respectively (Fig. 6). This indicates FANCD2 knockdown results in a reduction in number of viable cells in lung cancer.

MicroRNAs are found in a variety of body fluids including blood, saliva and urine, where they are quantifiable and extremely stable. For these reasons, miRNAs are excellent candidates as non-invasive biomarkers for early diagnosis of human disease, and for monitoring treatment response in cancer patients^52^. In our current research, the cells (A549 and H1299) and human tissues are from lung cancer patients. The miRNAs reported in this study are related to DNA homologous recombination repair deficiency, thus miRNA-200C and other FA related miRNAs are potential biomarkers of DNA repair deficiency in patients with lung cancer.

We noticed that the miRNA-200C expression was 1.44 folds higher in the A549D2D cell comparing to A549E cell (Table 1). We think this may be associated the incomplete FANCD2 knocking down in the A549 cell. Our knocking down strategy did not result in 100% ablation of FANCD2 expression. Therefore, it is possible that the partial FANCD2 expression was remained in the A549D2D cell. A small amount of the monoubiquitinated FANCD2 (the L-form of the FANCD2) remained in the A549D2D cells (Fig. 2), whereas the monoubiquitinated FANCD2 was absent from the H1299 D2D cell. Therefor we think the insignificant up-regulation of miR-200C in A549D2D cell may be caused by the incomplete knocking down of FANCD2 expression.

In addition to miR200C, other miRNAs (e.g. miRNA-26a, 143, 921, 184, 567, 873, 933, 1282 and 1288) were also found to be up-regulated, and a cluster of miRNAs (e.g. miRNA-20a, 20b, 129-3p and 262) were found to be downregulated in FANCD2 foci defective non-small cell lung cancer cells. It would be very interesting to study the biological function of these miRNAs, in particular the miRNA-20 (a and b), in regulation of cell proliferation in non-small cell lung cancers. In conclusion, the FA pathway regulates downstream genes through microRNAs in lung cancer and that MiRNA-200C appears to play a very important role in this regulation.

## Supplementary Information

Supplementary Information.
