# Supplementary material for: MiRNA-200C expression in Fanconi anemia pathway functionally deficient lung cancers
Source: Sci Rep. 2021 Feb 24;11:4420. doi: 10.1038/s41598-021-83884-9 (PMC7904768; doi:10.1038/s41598-021-83884-9)
Supplement: Supplementary file 1 — Supplementary Information. [file 41598_2021_83884_MOESM1_ESM.pdf]

MiRNA-200C Expression in Fanconi Anemia Pathway Functionally Deficient Lung Cancers

Wenrui Duan[1, 2,*], Shirley Tang[3], Li Gao[1], Kathleen Dotts[3], Andrew Fink[3], Arjun Kalvala[3], Brittany Aguila[3], Qi-En Wang[4], Miguel A Villalona-Calero[1,2*]

[1]Department of Human & Molecular Genetics, Herbert Wertheim College of Medicine, The Florida International University, Miami, Florida 33199

[2]Biomolecular Sciences Institute, The Florida International University, Miami, Florida 33199

[3]Comprehensive Cancer Center at The Ohio State University College of Medicine and Public Health, Columbus, Ohio 43210

[4]Department of Radiation Oncology, Comprehensive Cancer Center, The Ohio State University, Columbus, Ohio 43210

[*]Corresponding author

Wenrui Duan, Department of Human & Molecular Genetics, Herbert Wertheim College of Medicine, The Florida International University, Miami, Florida 33199

Tel. (305)-348-9135; E-mail, wduan@fiu.edu

or

Miguel A. Villalona-Calero, Miami Cancer Institute, 11015 SW 69th Avenue, Miami, Florida 33156

Tel. (786) 577-0577; E-mail, villalona.miguel@gmail.com

Original data for Fig. 2

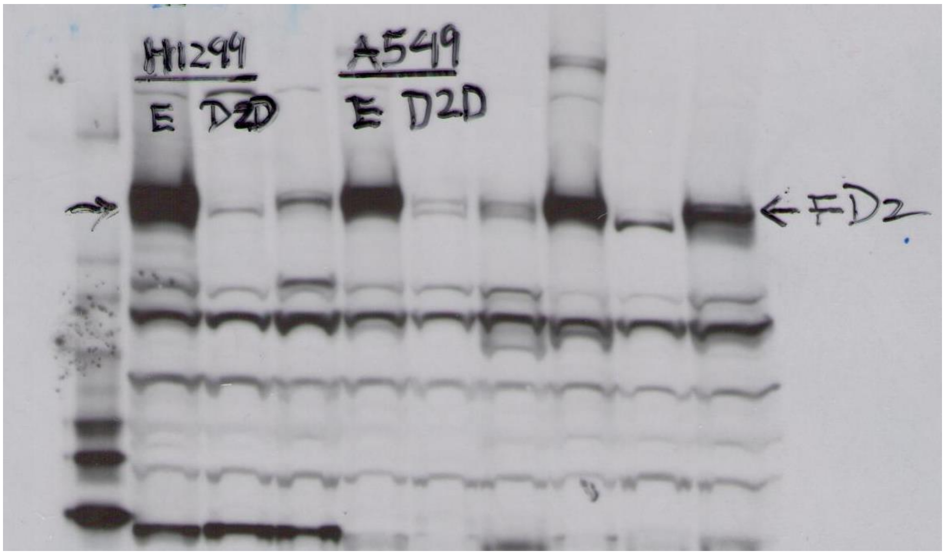

Lane 1 2 3 4 5 6 7 8 9 10

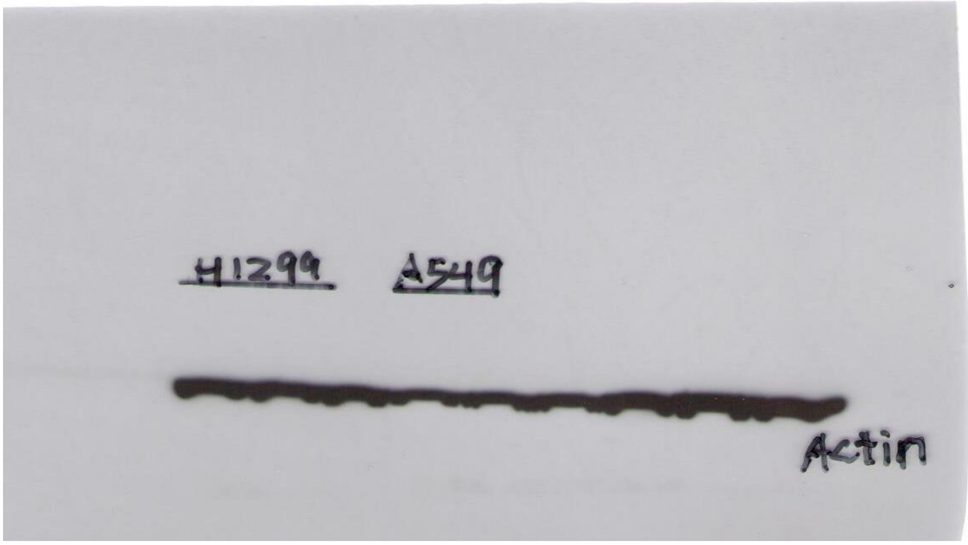

Lane 1 2 3 4 5 6 7 8 9 10

lane 1: protein ladder, lane 2: H1299E, lane 3: H1299D2D,
lane 5: A549E, lane 6: A549D2D
